# Why Persons Snore

**Published:** 1881-09

**Authors:** 


### WHY PERSONS SNORE.

SNORING is a noise made in the posterior part of the mouth and nasal fossæ during the moments of inspiration. It is due to a relaxation of the muscles in sleep by which they are left free to vibrate or flap in the two currents of air which enter at the same time through the nostrils and the mouth. Besides the vibration of the velum pendulum palati, or soft palate, there is also a vibration of the column of air itself. Thus is produced the rasping, snorting noise so well-known and so unpleasant to every one within earshot of the placid snorer himself.

A well-know physician was asked whether snoring is a disease.

"Not so much a disease as a bad habit," he said.

"Why do elderly or corpulent people commonly snore?"

"Because their systems are generally more relaxed in sleep, and their mouths then fall open. Any one will be likely to snore if he sleeps with his mouth open, and no one will if he shuts it."

"How can the habit be cured?"

"First, you must give a person a chance to breathe through the nose, and then make him do so. If there is any obstruction in the nasal passage that must be removed by treatment. Then if a snorer can't keep his mouth shut up by force of will, his jaw must be tied up. A harness for the lower jaw is sometimes employed in bad cases of snoring. A skull cap worn upon the head serves to hold a system of straps under the chin and keep the mouth shut until the patient can form a habit of sleeping on his side, or with his head sufficiently elevated to hold his jaw."

"Why is snoring, then, so common if it is so easily cured?"

"Because catarrhal troubles are so common, which prevent free inspiration through the nostrils. In sleeping-cars and in hotels one frequently hears the resonant snore, because people in those places usually go to sleep tired out. An old doctor used to advocate sleeping on the face to guard against the possibility of snoring."

---

"SEE her kiss that ugly dog," said one gentleman to a friend in a horse-car in a loud whisper, calling attention to a woman who was lavish with her endearments of a pet poodle. She overheard the comment, and glowering upon the unfortunate man said, in vinegary accents: "It won't hurt me, if I do." "Oh, I beg your pardon, madame, but my sympathy was wholly with the dog."

### ORIGIN OF THE RAILROAD BELL-ROPE.

In the early days of the railroad in this country the locomotive engineer was the master of the train. He ran it according to his judgment, and the conductor had very little voice in the matter. Collecting fares, superintending the loading and unloading of freight, and shouting "All aboard!" were all that the conductor was expected to do. The Erie Railway was then the New York & Erie Railroad. There was no railroad connection with Jersey City in 1842. Boats carried passengers from New York to Piermont-on-the-Hudson, which was then the eastern terminus of the road. Turner's, forty-seven miles from New York, was as far west as the railroad was in operation. One of the pioneer conductors of this line was the late Captain Ayres. He ran the only train then called for between the two terminal points. It was made up of freight and passenger cars. The idea of the engineer, without any knowledge of what was going on back of the locomotive, having his way as to how the train was to be run, did not strike the Captain as being according to the propriety of things. He frequently encountered a fractious passenger who insisted on riding without paying his fare. As there was no way of signaling the engineer, and the passenger could not be thrown from the train whlle it was in motion, the conductor in such cases had no choice but to let him ride until a regular stop was made. Captain Ayres finally determined to institute a new system in the running of trains. He procured a stout twine, sufficiently long to reach from the locomotive to the rear car. To the end of this string next the engineer he fastened a stick of wood. He ran this cord back over the cars to the last one.

That was the origin of the bell-rope, now one of the most important attachments of railroad trains. The idea was quickly adopted by the few roads then in operation, and the bell or gong in time took the place of the stick of wood to signal the engineer. Captain Ayres continued a conductor on this road under its different managers until he was superannuated and retired on a pension a year ago. He died a few months ago in Owego, N. Y., at the age of seventy-eight years.

---

Pleasures of hotel life: "Here's a fly in my soup, waiter." "Yes, sir; very sorry, sir; but you can throw the fly away and eat the soup, can't you?" "Of course I can. You didn't expect me to throw away the soup and eat the fly, did you?"

